# Optimizing functional, stability, and sensory attributes of quinoa beverages through bioprocessing, ultrasonication, and hydrocolloids

**DOI:** 10.1016/j.fochx.2025.102923

**Published:** 2025-08-18

**Authors:** Sobhy A. El-Sohaimy, Taha Mehany, Mohamed G. Shehata, Ashraf A. Zeitoun, Hanan M. Alharbi, Khairiah Mubarak Alwutayd, Mohamed A.M. Zeitoun

**Affiliations:** a Food Technology Department, Arid Lands Cultivation Research Institute, City of Scientific Research and Technological Applications, 21934 Alexandria, Egypt; b Department of Technology and Organization of Public Catering, Institute Sport, Tourism and Service, South Ural State University, 454080 Chelyabinsk, Russian Federation; c Food Research Section, Applied Research and Capacity Building Division, Abu Dhabi Agriculture and Food Safety Authority (ADAFSA), Abu Dhabi 20602, United Arab Emirates; d Department of Food Science, Faculty of Agriculture (Saba Basha), Alexandria University, 21531 Alexandria, Egypt; e Department of Biology, College of Science, Princess Nourah bint Abdulrahman University, P.O. Box 84428, Riyadh 11671, Saudi Arabia

**Keywords:** Antioxidant activity, Functional beverage, Saponin removal, Sensory, Beverage stability, Nutritional quality, Physicochemical, Plant-based milk

## Abstract

This study evaluated how various processing methods affect the nutritional, functional, and sensory qualities of a saponin-free quinoa-beverage. Bioprocessing techniques—soaking, germination, malting, and dehulling—were applied to reduce saponin and enhance nutritional value. Soaking eliminated saponins entirely, while germination and malting reduced levels to 0.02 % compared to 0.06 % in raw quinoa. These processes also improved functional properties such as water/oil absorption, foaming, and emulsification. Nutritional enhancements included increased polyphenol content, antioxidant activity, and higher levels of copper, manganese, and vitamin E. Ultrasonication at 15 min significantly improved suspension stability, especially when combined with 1.0 % xanthan gum, which maintained 100 % stability over eight days of cold storage. Sensory qualities were improved using natural additives like vanilla and guava. The optimal formulation used soaked quinoa, 15-min ultrasonication, 1.0 % xanthan gum, 1.0 % guava, and vanilla. This research presents a scalable method for producing a nutritious, stable, and appealing quinoa-based beverage for the functional drink market.

## Highlights

• Soaking completely eliminated saponins, improving taste and safety.
• Bioprocessing boosted polyphenols, antioxidant activity, and micronutrients including vitamin E, copper, and manganese.
• Enhanced water/oil absorption, foaming, and emulsifying properties were observed.
• Ultrasonication (15 min) with 1.0 % xanthan gum provided 100 % suspension stability for 8 days.
• Quinoa-guava drink with vanilla, ultrasonication & xanthan gum: nutritious, stable, and sensory appealing.

## Graphical abstract

**Figure:**
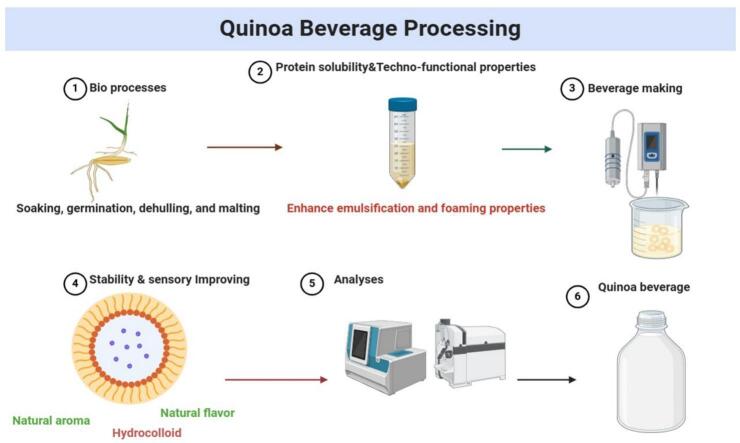

## 1 Introduction

Nowadays, healthy nutrition has become a vital aspect of society's demand, especially in relation to modern lifestyles, where wholesome food and a balanced diet are often neglected. As a result, it has become necessary to develop food products that can offer consumers appropriate, yet special and nutritious, beverages and foods (Fernandes et al., 2019). Recently, plant-based beverages have emerged as substitutes for food and dairy products, and there is an increasing trend in their consumption due to health reasons, such as milk allergies, stomach issues like lactose intolerance, and the desire to avoid cholesterol and antibiotic residues found in dairy milk (Mehany, Siddiqui, et al., 2023; Siddiqui et al., 2024; Youssef et al., 2020). Additionally, a wide range of plant-based beverages is available on the market, with soy milk being the most popular. Other options include beverages made from oats, rice, coconut, and almonds. However, many of these products have sensory attributes that consumers find unappealing, such as off-putting odors and flavors (Mäkinen et al., 2016). Milk alternatives derived from legumes, cereals, nuts, or modified cow's milk still face challenges in fully replacing traditional dairy. Some products trigger allergies (Fernández-Rivas & Asero, 2013), while others have a high glycemic index (Mendosa, 2008). Moreover, some commercial plant-based milk alternatives also contain additives such as sweeteners, which may reduce their overall nutritional quality (Mehany, Olawoye, & Popoola-Akinola, 2025). Despite these limitations, the dairy alternatives market continues to expand. The global dairy alternatives market size is estimated to reach USD 66.91 billion by 2030, registering a CAGR of 12.7 % from 2025 to 2030, according to a new report by Grand View Research, Inc. (Grandview Market Research, 2025). Furthermore, (Jeske et al., 2017) analyzed 17 commercial plant-based milk substitutes made from nuts, cereals, and legumes, revealing that many lacked sufficient protein content. Most were also highly unstable, prone to separation, making them inadequate replacements for cow's milk or even soy milk. While soy milk remains a leading alternative, it may not be the most sustainable option, as it does not thrive in cold climates and depends on extensive supply chains (Jeske et al., 2019). To meet consumer expectations, plant-based beverages must improve in both quality and functionality. Enhancing their sustainability and nutritional value is crucial for them to become viable dairy substitutes. As competition grows, research must address key challenges such as stability, sensory appeal, and functional properties, all while considering environmental and economic factors (Jeske et al., 2017). The physicochemical and nutritional characteristics of these products vary significantly based on their raw materials (Jeske, 2018). Therefore, there is a pressing need to develop new plant-based milk alternatives that are both nutritionally enhanced and free from adverse effects, offering improved sensory attributes.

Quinoa (*Chenopodium quinoa* Willd), a member of the Chenopodiaceae family, is widely recognized as a “superfood” and translates to “mother grain” in the Inca language. This edible seed is a stress-tolerant crop that has sustained Andean indigenous cultures for thousands of years, providing nutrition, sustenance, and medicinal benefits (Abdellatif, 2017; Graf et al., 2015; Ludena Urquizo et al., 2017; Vega-Gálvez et al., 2010). Interest in quinoa has surged due to its exceptional nutritional profile, which includes high-quality protein (Elsohaimy et al., 2015; Mäkinen et al., 2015), significant mineral and vitamin content, a low glycemic index, and lactose-free properties (El-Sohaimy et al., 2021; Sohaimy et al., 2018). The demand for quinoa is increasing in both developing and developed countries as consumers seek novel, nutrient-dense foods. As a gluten-free grain, quinoa serves as an excellent protein source for vegetarians and is a suitable alternative for individuals with gluten allergies or celiac disease (Ludena Urquizo et al., 2017; Mehany, Taha, et al., 2023; Zannini et al., 2018). Additionally, its low glycemic index makes it beneficial for preventing type 2 diabetes and an ideal replacement for common cereals in diabetic diets (Nanduri et al., 2019). While plant-based proteins generally have lower digestibility and often lack one or more essential amino acids (Friedman, 1996), quinoa stands out for its high protein content, superior quality, and functional properties. The Food and Agriculture Organization (FAO) notes that quinoa's protein composition is closer to the ideal balance than any other plant or cereal, rivaling the quality of milk protein (casein) (FAO, 2011). For this reason, quinoa is considered a complete food, offering a rich source of essential amino acids and making it an excellent candidate for developing plant-based milk alternatives (Abdellatif, 2017; Sharma et al., 2015).

Pineli et al. (2015) evaluated quinoa milk as a promising dairy milk alternative and found that it offers increased protein content, does not cause adverse effects in humans, and is beneficial for individuals with diabetes due to its low glycemic index. They also emphasized the need for further research on flavor enhancement techniques to improve consumer acceptance and satisfaction. Various processing methods, including soaking, germination, and malting, have been shown to enhance the nutritional value of cereals and pseudocereals while reducing anti-nutritional factors (Kaur & Tanwar, 2016). While quinoa's exceptional nutritional profile makes it a valuable ingredient in food products, many studies have noted its grassy and bitter off-flavors when used in high concentrations. Consequently, quinoa is often incorporated at lower levels to balance its taste (Mäkinen et al., 2016; Zannini et al., 2018). On the other hand, they still did not investigate the bioavailability of the protein and amino acids of the developed quinoa-based beverage, which needs more advanced research to confirm the health promoting properties of the developed beverage.

To our knowledge, no studies has yet been published on the development of a highly stable quinoa-based beverage with favorable sensory attributes. Given quinoa's well-documented health benefits, we hypothesize that this ancient pseudocereal could serve as a viable substitution to soy and other plant-based milk analogs. To expand quinoa's traditional applications and offer a healthier, more nutritious, and stable emulsion with excellent sensory acceptance, this study aims to develop a nutrient-rich quinoa-based oil/water beverage emulsion. Furthermore, seeking formulate it into a palatable and technologically optimized product suitable for large-scale industrial production.

## 2 Materials and methods

### 2.1 Materials

Quinoa seeds (*Chenopodium quinoa* Willd, variety: Chopaia) were obtained from the City of Scientific Research Farm in Burg El Arab city, Alexandria, Egypt, during the 2018/2019 season. The seeds were cleaned of impurities such as stones and leaves, then stored at were stored at −80 °C ± 2 °C for further processing and analysis. Arabic gum, xanthan gum, and psyllium husk were sourced from local markets in Alexandria and AWA Food Additives Company, Alexandria, Egypt. All chemicals and reagents used in this study were purchased from Sigma-Aldrich (St. Louis, Missouri, USA) and Aljumhouria Trades for Drugs and Chemicals (Alexandria, Egypt). They were of analytical grade and met the highest purity standards required.

### 2.2 Technological processing for the preparation of quinoa seeds

**Fig. 1:**
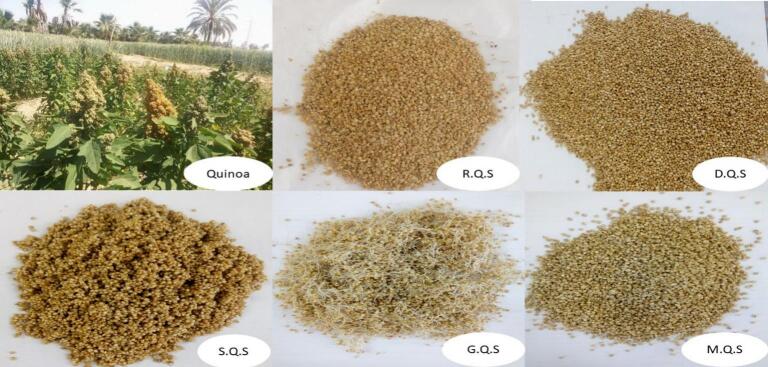
Quinoa: From plant to processed seed used in the experiments. Where: RQS: Raw quinoa seeds; DQS: Dehulled quinoa seeds; SQS: Soaked quinoa seeds; GQS: Germinated quinoa seeds; MQS: Malted quinoa seeds.

Fig. S1 presents the flow chart of quinoa seed preparation, which includes three developed steps: soaking in distilled water, germination, and malting of raw quinoa seeds. All treated seeds were compared with traditional dehulled quinoa seeds from the local market in Alexandria, Egypt. Additionally, Fig. 1 shows the process from the quinoa plant to processed seeds.

#### 2.2.1 Soaking process

Soaking of quinoa seeds was performed according to (Pineli et al., 2015) and (Kaur & Tanwar, 2016). Firstly, the raw quinoa seeds were washed with H_2_O with gentle agitation. This process was repeated three times at room temperature (25 ± 2 °C). The seeds were then soaked in distilled H_2_O for ten hours (the ratio of quinoa seeds to water was 1:5 *w*/*v*) at ambient temperature, with the water being changed every 3 h.

#### 2.2.2 Germination process

For the germination process, the method of Aguilar et al. (2001) was followed with minor adjustments. The raw quinoa seeds were soaked in distilled water for 10 h (1,5 *w/v*). They were then spread onto plastic trays covered with a wet, sterile cheesecloth and kept in a dark place at 25 °C for 72 h to allow germination. Once the sprout or hypocotyls reached a length of 7–10 mm, the germinated seeds were dried in a vacuum oven (VO-125, Seoul, Korea) at 40 °C for 4 h.

#### 2.2.3 Malting process

The malting process was adapted from Aguilar et al. (2001) with slight modifications. The germinated seeds from the previous stage were air-dried to remove excess moisture and then toasted in a vacuum oven (VO-125, Seoul, Korea) at 55 °C for 24 h. The radicles were manually removed to obtain malted quinoa seeds. Finally, the seeds were ground using a miller (Karizma-JX-1000 A, Karizma Appliances, New Delhi, India) and stored at 5 °C until analysis.

#### 2.2.4 Enhancement of protein solubility

Further, all treated quinoa seeds were steeped in a saline solution (0.03 M NaCl) acidified to pH 5 with citric acid, according to (Pineli et al., 2015) with slight modifications, to increase the protein content and its solubility in the beverage.

### 2.3 Proximate chemical analysis

The proximate composition of raw and treated quinoa samples was analyzed for ash, protein, fiber, lipid content, and energy value. Ash content was determined using a muffle furnace (Nabertherm L9/11/B180, Nabertherm GmbH, Lilienthal, Germany) at 550 °C until the sample turned gray. Protein content was calculated using the Kjeldahl method, with nitrogen content determined and multiplied by a factor of 6.25. Crude fiber content was assessed through digestion with dilute H₂SO₄ and KOH solutions in an ANKOM Fiber Analyzer (Model A200I, NY, USA). Lipid content was measured using the Folch method (Folch, Lees, & Sloane Stanley, 1957), employing a chloroform: methanol (2,1 *v*/*v*) solvent system with a 1:10 *w*/*v* seed-to-solvent ratio. The mixture was stirred for two hours, filtered, and the solvent was evaporated at 60 °C with vacuum. The nitrogen-free extract (NFE) was calculated by difference as follows:

(1) $$\mathrm{NFE}=100-\left(\mathrm{Protein}+\mathrm{Lipids}+\mathrm{Fiber}+\mathrm{Ash}\right)\;$$

Additionally, the gross energy (kcal/100 g DW) was determined, and all measurements of analyzed samples were made in triplicate.

### 2.4 Determination of saponins

Saponin determination was conducted using the standard Afrosymmetric method (Abdellatif, 2017; Ludena Urquizo et al., 2017). Quinoa seeds (0.5 g) were weighed and transferred to a test tube (15 cm in length and 15 mm in diameter), then 5 mL of deionized distilled water (Ultra Clear, SIEMENS UV-UFTM, Germany) were added to the tube. The capped tube was vigorously shaken for 30 s, then allowed to stand for 30 min before being shaken again for 30 s. This procedure was repeated twice, and after a 5-min rest, the foam height was measured. The saponin content of quinoa seeds, expressed as a percentage, was calculated according to the following equation:

(2) $$\mathrm{Ps}=\frac{\left(0.646\mathrm{xH}\right)-0.104\;}{\mathrm{Mx}10}\;$$

where: Ps: saponin content of quinoa, in percentage of mass. H: Foam height in (cm). M: Mass of the sample in (g).

### 2.5 Techno-functional characterization of quinoa seeds

#### 2.5.1 Determination of water and oil absorption

The approach described by (Sathe et al., 1982) was used to determine the oil and water absorption capacities of raw and treated quinoa seeds. Ground seed samples (1 g each) were blended with 10 mL of deionized H₂O and blended for 30 s using a blender (Waring commercial, USA). The mixture was left to stand at RT (25 °C) for half hour, then centrifuged at 4000 rpm for half hour. The supernatant volume was further recorded using a 10 mL cylinder. The same procedure was applied to determine the oil absorption capacity (OAC). The ability to absorb water or oil was expressed as the percentage of water or oil absorbed per gram of the sample.

#### 2.5.2 Foaming capacity and stability

The foaming characteristics of both untreated and processed quinoa seeds were assessed following the method described by Tsutsui (1988). A 1 % (*w*/*v*) quinoa suspension (20 mL) was homogenized at high speed (16,000 rpm) for 1 min using a laboratory blender to incorporate air. The resulting mixture was transferred into a 50 mL graduated cylinder, and the total volume was recorded immediately after blending to determine foam capacity (Fig. S2). To assess foam stability, the remaining volume was noted at 0.5, 5, 10, 20, 40, and 60 min. Foaming capacity was calculated using the following equation:

(3) $$\text{Foaming capacity}\%=\frac{\left(A-B\right)}{B}\times 100\;$$

where, A = volume after whipping (mL), and B = volume before whipping.

While foaming stability was calculated according to the following equation:

(4) $$\text{Foam stability}\;\left(\%\right)=\frac{\text{foam volume after time}\;\left(t\right)}{\text{Initial foam volume}}\times 100\;$$

#### 2.5.3 Emulsifying properties

The emulsion capacity and stability were evaluated following the procedure of (Yasumatsu et al., 1972). An emulsion was prepared by combining 1 g of quinoa sample with 10 mL of distilled water and 10 mL of soybean oil in a calibrated centrifuge tube. The mixture was centrifuged at 2000 rpm for 5 min, and emulsion capacity was determined by expressing the height of the emulsified layer as a percentage of the total mixture height (Fig. S3). To evaluate emulsion stability, the emulsion was heated at 80 °C for 30 min, then cooled under running tap water for 15 min, followed by a second centrifugation at 2000 rpm for 15 min. Emulsion stability was calculated as the proportion of the emulsified layer height to the total height of the mixture.

### 2.6 Extraction of bioactive components

To extract bioactive compounds from quinoa seeds, 80 % methanol was used as the solvent. One gram of both raw and treated quinoa seeds was combined with 10 mL of methanol (*w*/*v*) in a 100 mL conical flask. The mixture was stirred at 30 ± 1 °C for 3 h, then centrifuged at 8000 rpm. The solvent was subsequently evaporated from the supernatant, and the dried extract obtained was stored at 4 ± 1 °C (Kostić et al., 2019; Sonawane & Arya, 2013).

#### 2.6.1 Determination of Total phenolic content (TPC)

Total phenolic content (TPC) of quinoa extracts was determined using the Folin–Ciocalteu reagent, following the method described by (Kostić et al., 2019) The quinoa extract was mixed with methanol, centrifuged, and then reacted with Folin–Ciocalteu reagent, Na₂CO₃, and distilled water. After incubation in the dark, absorbance was measured at 650 nm using a T80 UV/VIS spectrometer (PG Instruments Ltd., Leicestershire, United Kingdom). A gallic acid standard curve (10–500 μg/mL, linear regression, y = 0.0003x + 0.015, R^2^ = 0.98) was used to calculate TPC, expressed as milligrams of gallic acid equivalents (GAE) per gram of dry weight.

#### 2.6.2 Determination of total flavonoids

The total flavonoid content of quinoa extract was measured using a colorimetric assay (Zhishen, Mengcheng, & Jianming, 1999). The extract was reacted with NaNO₂, AlCl₃, and NaOH, and the absorbance was measured at 510 nm. A catechol standard curve (5–100 μg/mL) was used to calculate the flavonoid content, which was expressed as μg catechol equivalents per gram of extract (μg/g).

#### 2.6.3 Antioxidant activity of quinoa extract by ABTS assay

The free radical scavenging activity of quinoa extract was measured using the ABTS assay (Arnao et al., 2019). ABTS and potassium persulfate solutions were mixed and allowed to react for 12 h, then diluted in methanol. The quinoa extract at various concentrations (5–100 μg/mL) was incubated with the ABTS solution, and absorbance was measured at 734 nm. A standard curve of ascorbic acid was used to calculate the scavenging capacity, expressed by the following formula:

(5) $$\%\mathrm{TAA}\;\left(\text{ABTS scavenging effect}\right)=\frac{A0-A1}{A0}\times 100\;$$

where: TAA is the total antioxidant activity, A₀ is the absorbance of the control reaction and *A₁* is the absorbance of the extract. All tests were performed in triplicate, and the results were averaged. The IC₅₀ value, which represents the concentration required to achieve 50 % antioxidant activity, was used to express the antioxidant capacity and compare it to that of ascorbic acid. IC₅₀ was determined by plotting antioxidant capacity (%) against extract concentration (μg/mL).

### 2.7 Nutritional characteristics of processed quinoa seeds

#### 2.7.1 Determination of minerals content

For mineral analysis, treated quinoa seeds (0.5 g) were ashed at 550 °C for 2 h, dissolved in 100 mL of 1 M HCl, and filtered. The dissolved ash was analyzed for minerals (iron, magnesium, potassium, sodium, zinc, manganese, copper, and calcium) using an ICP-MS (NEXION 300×, PerkinElmer Inc., Waltham, MA, USA). The mineral concentrations were determined in mg/100 g.

#### 2.7.2 Amino acid composition

Amino acid analysis of treated quinoa samples was conducted using an AAA 400 amino acid analyzer (INGOS Ltd., Czech Republic). The samples were hydrolyzed with 6 N HCl at 110 °C for 24 h, and excess HCl was removed under vacuum. The residue was dissolved in loading buffer, and the analysis was performed at 60 °C with a gas flow rate of 0.5 mL/min. Amino acid composition was calculated from the standard areas and expressed as mg/100 g of sample.

### 2.8 In vitro protein digestibility

First, protein was isolated from both raw and treated quinoa seeds following the procedure described by Elsohaimy et al. (2015). The resulting protein isolates were then used to assess in vitro protein digestibility. The digestibility analysis was carried out using a multi-enzyme method as described by Bodwell et al. (1980) and Carbonaro et al. (1997). Enzymatic digestion was performed using porcine pancreatic trypsin (type IX, 15,310 units/mg protein), bovine pancreatic chymotrypsin (type II, 48 units/mg solid), porcine intestinal peptidase (P-7500, 115 units/g solid), and bacterial protease (type XIV, 4.4 units/mg solid), all obtained from Sigma-Aldrich (St. Louis, MO, USA).

A 63.8 mg protein sample was dispersed in 10 mL of distilled water and equilibrated at 37 °C. The pH was adjusted to 8.0 using 1 N NaOH. Then, 1 mL of enzyme solution (containing 1.58 mg of trypsin, 3.65 mg of chymotrypsin, and 0.45 mg of peptidase) was added, and the mixture was incubated at 37 °C for 10 min. Subsequently, 1 mL of protease solution (1.48 mg) was added, and the digestion continued for an additional 9 min at 55 °C. After a final 1-min incubation at 37 °C, the pH was recorded and used to estimate the in vitro protein digestibility.

### 2.9 Quinoa beverage processing and optimization

**Fig. 2:**
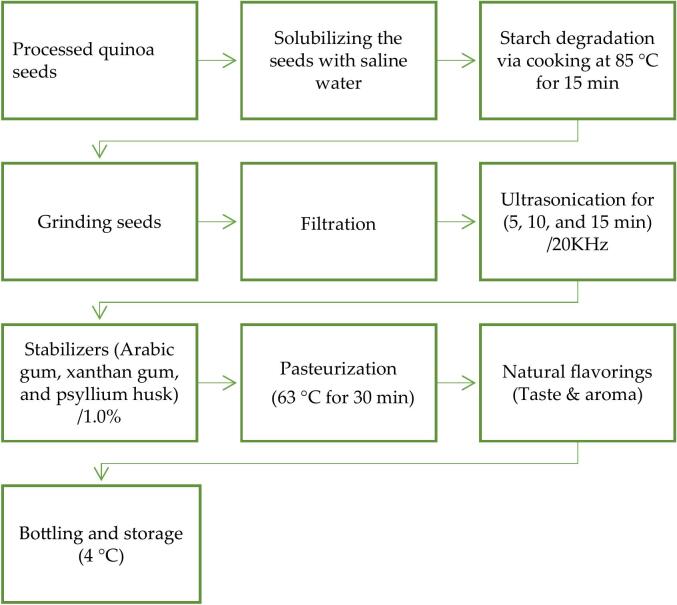
Processing of quinoa seeds for saponin removal enhances nutritional and functional properties by increasing protein content, leading to the development of a highly stable and sensorially appealing quinoa beverage.

The technological process for making quinoa milk was carried (Pineli et al., 2015), with modifications to improve the stability and sensorial properties. To prepare high quality quinoa beverage emulsion, in the current study; some modifications were applied in quinoa beverage processing to raise the nutritional value and remove the antinutritional factors in raw quinoa seeds, mainly saponins (Fig. 2). The treated quinoa seeds (soaked, germinated, malted and dehulled) were prepared for the optimization of the beverage emulsion. The treated quinoa seeds were cooked in saline (0.03 mol/L NaCl, pH 5} (1,7 *w*/*v*) at 100 °C for 10 min to allow starch cooking and degradation. The ratio (1,7), the experimentally designated lowest concentration required to prevent syneresis). The cooked samples were ground (trituration) in a semi-industrial blender (Robot Coupe Blixer 6 V.V., Robot Coupe, Burgundy, France) at high speed for 10 min, and the suspension was filtered through muslin cloth to obtain the beverages. Afterword, the quinoa beverage was ultrasonicated at varying time for 5, 10, and 15 min at 20 KHz. Ultrasound processing was performed using a probe-type ultrasonicator (Vibra Cell Sonics, model VCX 750, Newtown, Connecticut, USA) with a maximum net power output of 750 W, operating at a frequency of 20 kHz and an amplitude of 20 % (maximum amplitude: 40 %, corresponding to 228 μm). The treatment was applied using a 13 mm diameter titanium alloy probe. A thermocouple was used to monitor the sample temperature, which was carefully maintained below 30 ± 2 °C using an ice-water bath throughout the treatment. The sonicated process was applied to avoid sedimentation and maintaining the product completely uniform.

Three hydrocolloids were added for stabilization and improvement the organoleptic attributes (gum Arabic, psyllium Husk and xanthan gum) in ratio 1.0 %. The prepared quinoa beverage was bottled in sterilized glass bottles, pasteurized at 63 °C for 30 min in a water bath, rapidly cooled, and then stored at 4 °C for further analysis. We chose glass packaging because it better preserves product quality, prevents contamination, and ensures chemical stability compared to plastic. Additionally, glass is inert and recyclable, making it a more sustainable and safer option for beverage packaging.

### 2.10 Determination of physicochemical properties of quinoa beverage

#### 2.10.1 pH

The pH was measured using a pH meter (pH/MV & Temperature Meter, AD1030, Adwa Instruments, Szeged, Hungary).

#### 2.10.2 Viscosity

The apparent viscosity of quinoa beverage emulsions was measured using a rotational viscometer (STS-2011 SELECTA, Barcelona, Spain) equipped with a small sample adaptor (spindle no. 2) at 25 °C. A water bath was used for temperature control, and viscosity readings were taken at a rotational speed of 60 rpm.

#### 2.10.3 Total dissolved solids (TDS)

The TDS in quinoa beverage were determined using a digital refractometer (Hanna, HI 96811, Vöhringen, Germany) at 20 °C expressed as ^o^ Brix according to AOAC (2007).

#### 2.10.4 Suspension stability (syneresis)

To determine suspension stability, a slightly modified method suggested by Fernandes et al. (2019) was used. The quinoa beverage samples were poured in polyethylene terephthalate (PET) bottles and stored quiescently at 4 °C during the study. Visual suspension stability was made by looking for a demarcation line between upper and lower portions of the beverage after an 8-days period of quiescent storage at refrigerated temperature (4 °C). If a line of demarcation was observed, its height was measured, and the separation index was calculated as a ratio to total height of the beverage. The separation index would be 1 if no line was observed. The percentage separation was calculated as separation index multiplied by 100.

(6) $$\mathrm{Separation index}\;\left(\mathrm{SI}\right)=\mathrm{Ht}/H0$$

(7) Percent separation=SI∗100

where, Ht is the height of the lower phase at the interface after time “t” and H0 is the initial height of the beverage.

#### 2.10.5 Color analyses

The color of three optimized quinoa beverage samples was analyzed using a colorimeter (Smart Color Pro, Egypt). The L*, a*, and b* values were recorded, where L* indicates lightness (higher values for lighter colors), a* represents the red/green scale, and b* represents the yellow/blue scale. The instrument was calibrated before measurement to ensure accuracy.

### 2.11 Sensory evaluation

Sensory evaluation was carried out using a 9-point hedonic scale (Abd El-Aziz et al., 2024). The samples were coded and randomly distributed to each panelist. Each participant received 50 mL of the sample served at room temperature (30 °C). Attributes assessed included aroma, color, taste, mouthfeel (e.g., smoothness, thickness, body, grittiness), and overall preference. A total of 30 panelists, all possessing a background in food technology, compared control samples (without flavor additives) to those enriched with natural flavorings. The hedonic scale ranged from 1 (Dislike extremely) to 9 (Like extremely).

### 2.12 Statistical analysis

The data obtained were expressed as the mean ± standard deviation. All experiments were performed in triplicate. A one-way analysis of variance (ANOVA) was conducted, followed by Duncan's multiple range test using SPSS software (V16, Chicago, IL, USA). *P*-values less than 0.05 were considered statistically significant.

## 3 Results and discussion

### 3.1 A proximate chemical analysis of quinoa seeds

Chemical composition of raw and treated quinoa seeds is presented in Table 1. The pretreatment of quinoa seeds significantly affected their protein content. The germinating and malting process caused significant increase in protein content while soaking and dehulling decreased the protein content. The protein content in germinated seeds increased from 14.33 ± 0.10 to 15.21 ± 0.09 % and in malted seeds increased to 15.39 ± 0.22 % (*p* ≤ 0.05). Increasing of protein content by germination and malting might attributed to an increase in proteases and lowering of antinutritional factors which is leading to release soluble proteins. On the other hand, soaking and dehulling showed a negative impact on the protein content might be due to the removing part of protein content during soaking process (Table 1). Lipid content showed a slight decrease by soaking, germination and malting applied to quinoa seeds compared with raw seeds. Ash content showed a significant decrease by dehulling due to most of minerals accumulated in seeds husk (Table 1). Carbohydrates content was significantly decreased in all treatments compared to control due to the removing of soluble sugars and fibers during processes. The results of chemical analysis evident the potential benefits of malted and germinated quinoa seeds due to enhancing the essential nutritional elements (protein and minerals content). This agrees with the previous studies which stated that the malting process is an alternative to obtain high nutritional quality quinoa flour (Aguilar et al., 2001). Sprouting procedure might be considered a valid alternative to pearling or dehulling processes to improve the nutritional characteristics of quinoa seeds (Otterbach et al., 2021; Vega-Gálvez et al., 2010).

**Table 1:** Effect of processing technologies on proximate composition in quinoa seeds (on dry weight basis) g/100 g of sample.

Samples	Protein	Lipids	Ash	Crude Fiber	Carbohydrates
Raw seeds	14.33 ± 0.10^b^	6.76 ± 0.08^a^	1.98 ± 0.02^a^	2.86 ± 0.07^a^	73.55 ± 0.09^d^
Aqueous soaked seeds	13.97 ± 0.05^b^	6.46 ± 0.05^b^	1.75 ± 0.05^b^	1.93 ± 0.06^c^	71.88 ± 0.07^b^
Germinated seeds	15.21 ± 0.09^a^	6.13 ± 0.07^d^	1.86 ± 0.07^ab^	2.43 ± 0.08^b^	72.35 ± 0.04^c^
Malted seeds	15.39 ± 0.22^a^	6.43 ± 0.09^b^	1.87 ± 0.09^ab^	1.84 ± 0.08^c^	72.46 ± 0.19^c^
Dehulled seeds	12.34 ± 0.26^d^	6.13 ± 0.11^d^	1.15 ± 0.09^c^	1.44 ± 0.06^d^	70.47 ± 0.22^a^

Mean values with the different superscripts in the same column indicate a significant difference at *p* ≤ 0.05.

### 3.2 Saponins removal

Saponins are triterpenoid glucosides located in quinoa seeds' hull and responsible for the bitter taste of quinoa seeds and their products which making them undesirable for human consumption (Otterbach et al., 2021; Vega-Gálvez et al., 2010). The results in Table 2 revealed that soaking, malting and germination are effective strategies to eliminate saponins from quinoa seeds. Soaking and dehulling eliminate saponins while germination and malting were eliminated about 67 % of saponins from quinoa. The decrease in saponins is attributed to their leaching from quinoa seeds during soaking and washing treatments (El-Sohaimy et al., 2019). One of the most interesting findings from the current research, is that the soaking quinoa seeds in water for 2 h improving the quality of the seeds to be more proper for food applications.

**Table 2:** Effect of processing technologies on saponins content in quinoa seeds.

Samples	Saponin%
Raw seeds	0.06 ± 0.01^a^
Aqueous soaking	0.00 ± 0.01^c^
Germinated seeds	0.02 ± 0.00^b^
Malted seeds	0.02 ± 0.00^b^
De hulled seeds	0.01 ± 0.00^c^

Mean values with the different superscripts in the same column indicate a significant difference at *p* ≤ 0.05.

### 3.3 Minerals content

The mineral analysis demonstrated that dehulling removed about 46.82 % of mineral content (Table 3). The germinated quinoa seeds were considered the best treatment comparing with other treatments e.g., soaking, mating and dehulled seeds, which magnesium (22.38 ± 0.95), (280.7 ± 1.17), iron (10.80 ± 0.38), and copper (1.45 ± 0.07) mg/L. In all, the minerals content of all treated quinoa seeds was less than its content in raw seeds. In addition, during germination, and malting processes many minerals are required as coenzymes for carbohydrate and protein hydrolysis, which are moved to the seed's radicles (Suárez-Estrella et al., 2020). We can notice the higher loss in minerals shown in pearled seeds due to most of the ash and minerals are in external layer or quinoa seeds hull. Previous research has shown that quinoa minerals are concentrated in the outer bran layers, with levels higher than those found in other cereals (Prego et al., 1998). This data suggests that quinoa seed flour contains a more balanced and superior profile of essential minerals compared to other cereals.

**Table 3:** Minerals content of processed quinoa seeds (mg/100 g).

Minerals	Raw seeds	Soaked seeds	Germinated seeds	Malted seeds	Dehulled seeds
Calcium (Ca)	48.10 ± 1.85^a^	46.19 ± 1.02^a^	42.54 ± 1.2^b^	40.24 ± 1.01^b^	22.52 ± 1.50^c^
Copper (Cu)	1.82 ± 0.21^a^	1.54 ± 0.08^b^	1.45 ± 0.07^b^	1.55 ± 0.08^b^	1.05 ± 0.08^c^
Iron (Fe)	14.25 ± 1.02^a^	10.84 ± 0.36^b^	10.80 ± 0.38^b^	10.22 ± 0.86^b^	7.86 ± 0.37^c^
Magnesium (Mg)	24.43 ± 1.00^a^	18.85 ± 0.62^c^	22.38 ± 0.95^b^	19.55 ± 0.60^c^	10.25 ± 0.44^d^
Manganese (Mn)	0.24 ± 0.01^a^	0.22 ± 0.01^b^	0.19 ± 0.01^c^	0.16 ± 0.01^d^	0.02 ± 0.00^e^
Sodium (Na)	20.25 ± 0.77^a^	14.17 ± 0.43^b^	14.52 ± 0.44^b^	13.92 ± 0.33^b^	8.86 ± 0.53^c^
Potassium (K)	133.37 ± 1.81^a^	68.88 ± 1.56^c^	80.50 ± 1.23^b^	65.68 ± 0.95^d^	29.51 ± 0.88^e^
Zinc (Zn)	0.18 ± 0.01^a^	0.14 ± 0.01^b^	0.14 ± 0.01^b^	0.14 ± 0.00^b^	0.08 ± 0.00^c^

Mean values with the different superscripts in the same row indicate a significant difference at p ≤ 0.05. Where: R raw seeds, S = soaked seeds, G = germinated seeds, M = malted seeds, D = dehulled seeds.

### 3.4 Amino acids composition

The amino acid contents of the raw and treated quinoa seeds through soaking, germination, malting, and dehulling is shown in Table 4. We can notice that threonine, histidine, and lysine in raw seeds were significantly greater than all treated shambles. The malted seeds have more content of valine, methionine, isoleucine, leucine, phenylalanine than raw which recorded 3.25 ± 0.04 1.50 ± 0.06, 2.93 ± 0.08, 5.51 ± 0.16, and 4.22 ± 0.03 (mg/100 g) respectively, and this ratio is higher than samples like soaked, and germinated. Table 4 also illustrated that the content of non-essential amino acids was slightly greater than treated quinoa seeds.

**Table 4:** Amino acids composition of processed quinoa seeds (mg/100 g).

Amino acids	Raw seeds	Soaked seeds	Germinated seeds	Malted seeds	Dehulled seeds
Essential					
Threonine	3.25 ± 0.05^a^	2.89 ± 0.07^bc^	2.75 ± 0.02^c^	3.07 ± 0.09^ab^	2.55 ± 0.21^d^
Cystine	1.96 ± 0.06^b^	1.82 ± 0.05^c^	2.10 ± 0.02^a^	1.80 ± 0.03^c^	1.36 ± 0.04^d^
Valine	3.17 ± 0.04^b^	3.27 ± 0.04^b^	2.99 ± 0.11^c^	3.25 ± 0.04^a^	3.18 ± 0.14^b^
Methionine	1.32 ± 0.07^b^	1.35 ± 0.03^b^	1.41 ± 0.02^ab^	1.50 ± 0.06^a^	1.32 ± 0.09^b^
Isoleucine	2.52 ± 0.04^b^	2.82 ± 0.05^a^	2.47 ± 0.10^b^	2.93 ± 0.08^a^	2.18 ± 0.04^c^
Leucine	5.30 ± 0.16^ab^	5.12 ± 0.09^bc^	4.95 ± 0.07^c^	5.51 ± 0.16^a^	4.95 ± 0.08^c^
Phenylalanine	4.14 ± 0.09^a^	4.15 ± 0.15^a^	3.81 ± 0.06^b^	4.22 ± 0.03^a^	4.18 ± 0.02^a^
Histidine	3.81 ± 0.02^a^	3.51 ± 0.04^b^	3.20 ± 0.09^c^	3.45 ± 0.06^b^	3.12 ± 0.07^c^
Lysine	4.53 ± 0.05^a^	4.32 ± 0.09^b^	3.63 ± 0.14^d^	4.43 ± 0.05^ab^	4.09 ± 0.06^c^
Non-essential					
Aspartic acid	9.89 ± 0.12^a^	8.69 ± 0.19^b^	8.33 ± 0.08^c^	8.67 ± 0.28^b^	7.76 ± 0.08^d^
Serine	3.61 ± 0.03^a^	3.15 ± 0.08^c^	2.81 ± 0.03^d^	3.45 ± 0.13^a^	3.26 ± 0.11^c^
Glutamic acid	14.57 ± 0.25^a^	13.61 ± 0.33^b^	11.65 ± 0.53^d^	13.50 ± 0.06^bc^	13.03 ± 0.05^c^
Proline	7.15 ± 0.12^a^	6.62 ± 0.49^b^	6.15 ± 0.16 ^c^	6.10 ± 0.05^c^	2.67 ± 0.10^d^
Glycine	5.49 ± 0.02^a^	5.16 ± 0.16^b^	4.35 ± 0.06^d^	4.74 ± 0.07^c^	5.16 ± 0.15^b^
Alanine	4.81 ± 0.05^a^	4.45 ± 0.07^b^	4.22 ± 0.09^c^	4.75 ± 0.09^a^	4.05 ± 0.07^d^
Tyrosine	1.49 ± 0.03^d^	1.61 ± 0.07^c^	1.92 ± 0.04^b^	2.21 ± 0.12^a^	1.63 ± 0.08^c^
Arginine	6.91 ± 0.08^b^	9.76 ± 0.05^a^	5.42 ± 0.08^e^	6.44 ± 0.10^c^	5.61 ± 0.07^d^

Mean values with the different subscripts in the same row indicate a significant difference at p ≤ 0.05.

### 3.5 Total phenolic and flavonoids content

The present study revealed that the TPC in the raw quinoa seeds extract was 317.27 ± 3.68 μg GAE/g and there is a significant increase (*p* < 0.05) in TPC in all treatments which was 376.45 ± 4.51, 341.57 ± 3.37, 353.40 ± 1.73, 364.96 ± 4.98 μg GAE/mg in soaked seeds, germinated seeds, malted seeds, and dehulled seeds respectively (Table 5). Generally, quinoa seeds are considered rich sources of polyphenolic compounds that can enhance the nutritional properties of foods, such as beverage emulsions. Additionally, the total flavonoid content was significantly higher in malting and dehulling samples compared to soaked and germinated ones. The observed increase in the level of phenolic compounds during germination and soaking might be due to the freeing of phenolic compounds bound to the seed matrix under the effect of esterases synthesized during the process (Ogungbenle, 2003). Moreover, the temperature of malting up to 55 °C causes changes in the cell wall structure provoking increase in the total phenolic content due to the release of glycosylated and esterified phenolic compounds (Ogungbenle et al., 2009).

**Table 5:** Effect of processing technologies on phytonutrients composition in quinoa extract.

Samples	TPC (μg GAE/g)	TFC (μg CE/g)
Raw seeds	317.27 ± 3.68^e^	11.62 ± 0.47^c^
Soaked seeds	376.45 ± 4.51^a^	9.69 ± 0.30^d^
Germinated seeds	341.57 ± 3.37^d^	9.26 ± 0.24^d^
Malted seeds	353.40 ± 1.73^c^	14.86 ± 0.33^a^
Dehulled seeds	364.96 ± 4.98^b^	13.31 ± 0.38^b^

Mean values with the different superscripts in the same row indicate a significant difference at p ≤ 0.05.

### 3.6 Antioxidant activity

The antioxidant capacity via the ABTS method was estimated for the five samples compared to ascorbic acid. As shown in Table 6, the scavenging capacity was significantly increased by the malting process. It is worth noting that all quinoa seed samples exhibited strong antioxidant activity, with % inhibition values of 98.90 ± 0.47 (raw seeds), 98.56 ± 0.19 (soaked seeds), 91.73 ± 0.14 (germinated seeds), 97.28 ± 0.17 (malted seeds), and 97.42 ± 0.39 (dehulled seeds). The inhibition concentration (IC_50_), which represents the concentration required to achieve 50 % inhibition capacity, is commonly used to express antioxidant activity and compare the antioxidant capacity of ascorbic acid. IC_50_ values were determined from a plot of antioxidant capacity (%) against extract concentration (μg/mL). IC_50_ of soaked and germinated seeds have recorded (11.35 ± 0.04 and 11.31 ± 0.08 respectively) and comparable with the ascorbic acid which had IC_50_ (10.81 ± 0.06) that used as standard. The high antioxidant activity of all processed quinoa seeds is related to with their total phenolic and flavonoid content. These results are in agreement with the previous study by Suárez-Estrella et al. (2020).

**Table 6:** Effect of processing technologies on antioxidants activity in quinoa extracts.

Concentration of Samples (μg/mL)	% Inhibition of ABTS					
	R.S	S. S	G. S	M.S	D·S	Ascorbic acid
5	22.34 ± 0.31^d^	25.68 ± 0.48^b^	20.53 ± 0.57^e^	21.47 ± 0.63^c^	25.12 ± 0.49^b^	38.15 ± 0.26^a^
10	35.42 ± 0.58^d^	39.63 ± 0.44^b^	31.57 ± 0.61^e^	33.11 ± 0.80^c^	38.96 ± 0.53^b^	58.42 ± 0.33^a^
15	48.67 ± 0.49^d^	52.68 ± 0.32^b^	46.53 ± 0.40^e^	47.53 ± 0.54^c^	51.68 ± 0.44^b^	69.15 ± 0.28^a^
20	78.67 ± 0.49^d^	87.68 ± 0.44^b^	81.53 ± 0.96^c^	81.05 ± 0.52^c^	87.88 ± 0.25^b^	92.15 ± 0.28^a^
40	87.82 ± 0.32^d^	95.76 ± 0.57^a^	82.68 ± 0.40^e^	87.89 ± 0.63^d^	90.99 ± 0.64^c^	93.85 ± 0.39^b^
60	96.16 ± 0.46^b^	97.36 ± 0.34^a^	85.18 ± 0.59^d^	91.78 ± 0.30^c^	92.07 ± 0.11^c^	95.43 ± 0.51^b^
80	98.84 ± 0.62^a^	98.35 ± 0.29^a^	87.72 ± 0.39^d^	96.34 ± 0.46^b^	94.62 ± 0.38^c^	98.61 ± 0.41^a^
100	98.90 ± 0.47^ab^	98.56 ± 0.19^b^	91.73 ± 0.14^d^	97.28 ± 0.17^c^	97.42 ± 0.39^c^	99.47 ± 0.46^a^
IC_50_	12.54 ± 0.09^a^	11.35 ± 0.04^d^	12.22 ± 0.07^c^	11.31 ± 0.08^d^	12.34 ± 0.03^b^	10.81 ± 0.06^e^

Mean values with the different superscripts in the same row indicate a significant difference at p ≤ 0.05.Where: R raw seeds, S = soaked seeds, G = germinated seeds, M = malted seeds, D = dehulled seeds.

Although TPC is often used as a general indicator of antioxidant potential, it does not always directly correlate with measured antioxidant activity. In this study, while the soaked and dehulled quinoa extracts exhibited the highest TPC values (376.45 and 364.96 μg GAE/g, respectively), they also showed the strongest ABTS radical scavenging activity (IC₅₀: 11.35 and 12.34 μg/mL). Interestingly, malted seeds, with slightly lower TPC (353.40 μg GAE/g), demonstrated comparable antioxidant activity (IC₅₀: 11.31 μg/mL), likely due to their significantly higher flavonoid content (14.86 μg CE/g).

In this study, the slightly lower antioxidant activity observed in the germinated sample (91.73 %) compared to the soaked sample (98.56 %) may be attributed not only to differences in TPC values but also to the qualitative composition of phenolics, presence of non-phenolic antioxidants, or matrix interactions that influence radical scavenging efficiency. This highlights the importance of considering both the quantity and quality of antioxidant compounds when interpreting antioxidant behavior.

These findings support the notion that antioxidant activity is influenced not only by the quantity but also by the quality and bioavailability of phenolic compounds, as well as the possible contribution of other non-phenolic antioxidants generated during processing. This underscores the importance of comprehensive profiling, beyond TPC alone, when evaluating the antioxidant behavior of food matrices.

### 3.7 In vitro protein digestibility

Before incorporating the treated quinoa seeds into the beverage formulation, we assessed the in vitro protein digestibility of each sample to evaluate the effects of processing on protein quality. The treatments included raw (control), soaked, germinated, malted, and dehulled quinoa seeds. The protein digestibility values were as follows: raw (control): 77.15 ± 1.02 %, soaked: 80.38 ± 0.95 %, germinated: 82.36 ± 1.10 %, malted: 84.37 ± 1.08 %, and dehulled: 87.69 ± 1.12 %.

These results demonstrate a progressive improvement in protein digestibility with each processing step, indicating enhanced bioavailability of quinoa proteins. The particularly high digestibility observed in the malted and dehulled samples highlights their potential to enhance the nutritional functionality of quinoa-based beverages.

Our findings are consistent with previous research, which reported quinoa protein digestibility values ranging from 75.3 % to 84 % (Repo-Carrasco-Valencia & Serna, 2011; Zia-Ur-Rehman and Shah, 2001), and significantly higher than those reported for wheat, typically between 47 % and 59 % (Căpriţă et al., 2012; Elsohaimy et al., 2015). Consequently, each processed quinoa seed type was subsequently used as a distinct ingredient in the development of the quinoa-based beverage.

Recent studies also align with our findings, particularly regarding raw quinoa seeds. Ruales & Nair (1994) reported that the in vitro protein digestibility of raw quinoa, measured via enzymatic methods, was 78 %, significantly lower (*P* > 0.01) than that of casein (91 %) and slightly lower than that of raw washed quinoa (83 %). Their study further showed that the removal of the saponin-rich outer seed layers improved protein digestibility by 7 % (P > 0.01), and heat treatments also enhanced digestibility compared to raw samples. However, a 60-min cooking treatment slightly reduced protein digestibility to 77 %.

Moreover, our highest measured value (87.69 %) closely aligns with another recent study (Li et al., 2025) that reported an in vitro protein digestibility of 89.21 % for a quinoa-based protein beverage, further supporting quinoa's potential as a high-quality protein source.

### 3.8 Functional properties of quinoa flour

#### 3.8.1 Water and oil absorption, emulsion capacity and stability

Water and oil absorption of beverage are crucial techno-functional property due to improving mouthfeel and flavor retention in final product. In Table 7A, processed quinoa seeds showed higher score of water absorption and recorded ratios 32.62 ± 0.34, 27.52 ± 0.56, 27.48 ± 0.50, 26.95 ± 0.06 % for soaked, germinated, malted and dehulled seeds, respectively than that of raw quinoa seeds (25.78 ± 0.75 %). The protein content in viscous foods, such as soups, dough, and baked goods, affects water absorption, making quinoa flour an ideal ingredient for these formulations (Mehany, Taha, et al., 2023). The same trend was noted for oil absorption as well. The oil absorption capacity of quinoa seed flour (46 %) (Nair et al., 2020) was lower than that observed in the current study. Emulsion properties are key functional attributes that significantly impact the behavior of food products. The average emulsion capacity of raw quinoa seeds was 54.82 ± 0.65 %, while emulsion stability averaged 40.03 ± 0.17 %. Soaked quinoa seeds showed the highest emulsion capacity (60.02 ± 0.50 %) and stability (52.40 ± 1.27 %). These values surpass those of yam bean dehulled seed flours (10–20 %), soy flour (18 %) (Taha et al., 2024), and pigeon pea (49.9 %). This indicates that quinoa seeds could serve as an excellent alternative to various cereals and grains in food formulations, particularly as binders and stabilizers for colloidal foods.

**Table 7:** Effect of processing technologies on techno-functional characteristics of processed quinoa seeds: (A) Water and oils absorption and emulsifying properties; (B) Foaming properties.

A				
Samples	Water absorption %	Oil absorption	Emulsifying capacity %	Emulsifying stability %
Raw seeds	25.78 ± 0.75^c^	13.96 ± 0.08^c^	54.82 ± 0.65^b^	40.03 ± 0.17^d^
Aqueous soaking	32.62 ± 0.34^a^	15.29 ± 0.28^b^	60.02 ± 0.50^a^	52.40 ± 1.27^a^
Germinated seeds	27.52 ± 0.56^b^	15.26 ± 0.34^b^	54.59 ± 1.19^b^	50.30 ± 0.80^b^
Malted seeds	27.48 ± 0.50^b^	15.37 ± 0.35^b^	51.24 ± 1.19^d^	52.81 ± 0.46^a^
Dehulled seeds	26.95 ± 0.06^b^	15.95 ± 0.07^a^	51.57 ± 1.30^c^	44.18 ± 0.87^c^
B				

Samples	Foaming capacity%	Foaming stability (%) at time intervals					
		0.5 min	5 min	10 min	20 min	40 min	60 min
Raw seeds	21.22 ± 1.10^c^	75.57 ± 0.63^b^	25.54 ± 0.62^c^	0.00 ± 0.00^b^	0.00 ± 0.00^d^	0.00 ± 0.00^c^	0.00 ± 0.00^d^
Aqueous soaking	40.63 ± 0.89^a^	99.82 ± 0.22^a^	99.62 ± 0.35^a^	99.41 ± 0.52^a^	86.37 ± 1.08^b^	74.61 ± 0.38^a^	49.62 ± 0.37^c^
Germinated seeds	40.80 ± 0.78^a^	99.81 ± 0.19^a^	99.69 ± 0.32^a^	98.72 ± 1.11^a^	87.67 ± 0.48^a^	74.56 ± 0.52^a^	51.19 ± 1.16^b^
Malted seeds	35.77 ± 0.78^b^	99.71 ± 0.28^a^	99.61 ± 0.35^a^	99.53 ± 0.50^a^	84.87 ± 0.79^c^	70.97 ± 0.66^b^	56.63 ± 0.67^a^
Dehulled seeds	10.61 ± 0.63^d^	50.29 ± 0.84^c^	29.76 ± 0.65^b^	0.00 ± 0.00^b^	0.00 ± 0.00^d^	0.00 ± 0.00^c^	0.00 ± 0.00^d^

Mean values with the different superscripts in the same column indicate a significant difference at p ≤ 0.05.

#### 3.8.2 Foaming capacity and stability

Foaming capacity and stability are important factors in evaluating the functional properties of food products (Elsohaimy et al., 2015; Mäkinen et al., 2015). As shown in Table 7B, the foaming capacity of untreated quinoa seeds was 21.22 ± 1.10 %, and its stability was 0.00 ± 0.00 % after 60 min. The foaming capacity of soaked, germinated, and malted seeds quinoa seeds flour were 40.63 ± 0.89 % and 40.80 ± 0.78 %, 35.77 ± 0.78 respectively, and recorded the highest stability after 60 min. However, the dehulled seeds had the lowest foaming capacity (10.61 ± 0.63 %) and stability (0.00 ± 0.00 %).

### 3.9 pH, viscosity and color properties of quinoa beverages

**Fig. 3:**
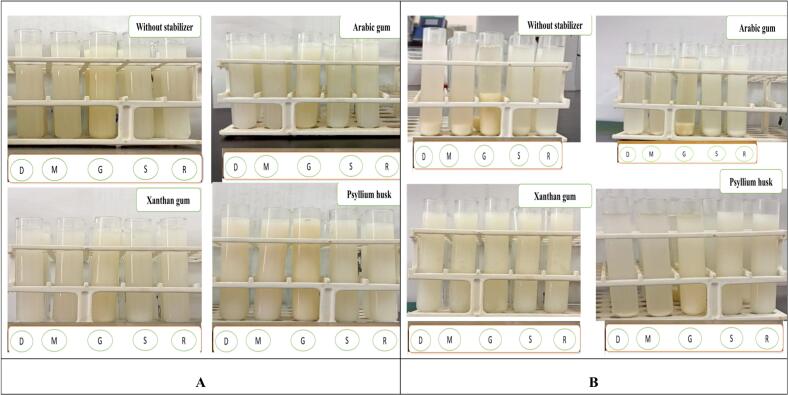
(A) Quinoa beverage at zero day (processing day) using three types of stabilizers. Where: R = raw beverage, S = soaked beverage, G = germinated beverage, M = malted beverage, D = dehulled beverage. (B) Quinoa beverage after 8 days of storage using three types of stabilizers. Where: R = raw beverage, S = soaked beverage, G = germinated beverage, M = malted beverage, D = dehulled beverage.

The obtained findings in Table 8 presents the effect of processing on the physicochemical properties of quinoa beverages. It is evident from the table that the pH remained largely unaffected overall, with a slight decrease observed in the soaked, germinated, malted, and dehulled quinoa beverages. Additionally, the viscosity of all treated quinoa beverages showed a slight increase compared to the control. This increase in viscosity can be attributed to the soaking, germination, and malting processes, which enhance the fragility of the quinoa kernels, making them easier to break during the grinding process. This result was in agreement with those data of final malted millet milk studied by (Nair et al., 2020). The color in raw beverage is lighter than all treatments and that owing to the different processing could resulted the metabolic interactions and that occurred of dark color of the end products (Fig. 3A). Malted beverages showed less greenness (lower a*) than the raw beverage and most yellowness (lower b*). Soaked beverage showed lighter (higher l*) comparable with raw beverage.

**Table 8:** pH, viscosity and color properties of treated quinoa beverage.

Physicochemical properties		Raw beverage	Soaked beverage	Germinated beverage	Malted beverage	Dehulled beverage
pH		5.43	5.11	4.72	5.22	5.31
Viscosity (cP)		20.45	23.12	21.14	22.56	21.04
Color values⁎	l	99.00	98.00	97.00	94.86	92.86
	a	−11.76	−10.76	−14.48	−4.25	−4.33
	b	−61.06	−66.06	−60.13	−69.01	−71.83

⁎ l is lightness: 100 is whiter, and 0 is dark.; a is redness: −100 is green, and + 100 is red; b is yellowness: −100 is blue, and 100+ is yellow.

### 3.10 Suspension stability of quinoa beverages

The food industry is increasingly embracing green technologies to enhance sustainability and efficiency. Ultrasound, as an environmentally friendly processing technique, holds significant potential in food applications by enhancing extraction efficiency, product stability, and the preservation of nutritional and sensory qualities. It also contributes to shelf-life extension and reduces the reliance on food additives (Taha et al., 2024). The method is based on the cavitation phenomenon, which occurs due to the alternating compression and expansion phases induced by ultrasonic waves ranging as they pass through the substance (Ali et al., 2022). The effect of ultra-sonication (20 KHz) at varying times on stability properties of quinoa beverages was investigated. Both raw and treated quinoa beverages was more stable by gradually increasing of sonication time at 15 min. The emulsion syneresis of quinoa beverages decreased due to sonication treatment with respect to treatment time. It was previously observed that the sonication treatment had synergetic effect on physicochemical properties and obtained high stable coconut milk (Indu et al., 2019). Table 9 shows the effect of ultrasonication time on suspension stability of treated quinoa beverage at zero day. In the current study the ultrasonication technique is not enough to obtain 100 % stable final quinoa beverages. Subsequently, the addition of some food stabilizers was investigated to explore its effect on stability along with storage period. The impact of numerous stabilizers on the suspension stability and syneresis phenomena of quinoa beverages are illustrated in Table 10. There was no syneresis % at the zero day or processing day in all quinoa beverages and scored 100 % of stability. The raw and soaked quinoa beverages containing Arabic gum exhibited a suspension stability ratio of 100.0 %, with the mixture remaining uniform and delicate. However, after 8 days of cold storage, the results indicated that among the different stabilizers (arabic gum, psyllium husk, and xanthan gum), only xanthan gum showed 0 % syneresis, ensuring the beverage remained stable (Fig. 3B). Moreover, xanthan gum proved to be an effective stabilizer for improving the suspension stability of quinoa-based beverages. This is due to its ability to form a strong physical network within the system, resisting gravitational forces and preventing particle settling. This finding aligns with the work of (Luo et al., 2020) who optimized the formula and processing of a sweet potato leaf powder-based beverage and concluded that xanthan gum effectively improves suspension stability, outperforming other stabilizers such as guar gum, arabic gum, hydroxypropyl methylcellulose, carboxymethylcellulose sodium, sodium alginate, and konjac gum.

**Table 9:** Effect of ultrasonication time on suspension stability of treated quinoa beverage at zero day.

Quinoa beverage type	Time of sonication (min)	Suspension stability %	Syneresis %
Raw seeds-based-beverage	0	75	25
	5	80	20
	10	86	14
	15	90	10
Soaked seeds-based-beverage	0	76	24
	5	82	18
	10	85	15
	15	92	8
Germinated seeds-based- beverage	0	76	24
	5	79	21
	10	81	19
	15	90	10
Malted seeds-based-beverage	0	74	26
	5	80	20
	10	84	16
	15	89	11
Dehulled seeds-based-beverage	0	75	25
	5	78	22
	10	85	15
	15	88	12

**Table 10:** Effect of various types of hydrocolloids on suspension stability and syneresis of quinoa beverages after 8 days of cold storage (4 °C).

Quinoa Beverage Type	Hydrocolloids Type (1 %)	Days of Storage	Suspension Stability %	Syneresis %
Raw seeds-based-Beverage	Without	0	100	0
	Arabic gum		100	0
	Psyllium husk		100	0
	Xanthan gum		100	0
	Without	8	92.31	7.69
	Arabic gum		100	0
	Psyllium husk		93.84	6.15
	Xanthan gum		100	0
Soaked seeds-based-Beverage	Without	0	100	0
	Arabic gum		100	0
	Psyllium husk		100	0
	Xanthan gum		100	0
	Without	8	90.79	9.24
	Arabic gum		100	0
	Psyllium husk		89.23	10.76
	Xanthan gum		100	0
Germinated seeds-based-Beverage	Without		100	0
	Arabic gum		100	0
	Psyllium husk		100	0
	Xanthan gum		100	0
	Without	8	53.84	46.16
	Arabic gum		81.53	18.46
	Psyllium husk		84.61	15.38
	Xanthan gum		100	0
Malted seeds-based-Beverage	Without	0	100	0
	Arabic gum		100	0
	Psyllium husk		100	0
	Xanthan gum		100	0
	Without	8	87.69	12.30
	Arabic gum		96.92	3.07
	Psyllium husk		98.46	1.53
	Xanthan gum		100	0
Dehulled seeds-based-Beverage	Without	0	100	0
	Arabic gum		100	0
	Psyllium husk		100	0
	Xanthan gum		100	0
	Without	8	87.69	12.30
	Arabic gum		96.93	3.03
	Psyllium husk		98.46	1.53
	Xanthan gum		100	0

Xanthan gum provided 100 % suspension stability due to its unique ability to form a highly viscous and elastic network within the beverage matrix, effectively preventing particle aggregation and sedimentation. Its shear-thinning behavior ensures uniform dispersion under mixing while maintaining structural integrity during storage, which makes it superior in maintaining homogeneity and preventing syneresis over extended periods.

Our results align well with those reported by Luo, Mu, Sun and Chen (2020), who developed and optimized a sweet potato leaf powder (SPLP)-based beverage with enhanced suspension stability and nutritional value. They found that the ultrafine SPLP exhibited a narrower particle size distribution and higher suspension stability, especially when combined with 2.5 % xanthan gum as a stabilizer. Their optimized formula, which included xanthan gum alongside other functional additives, resulted in a beverage with improved flavor, texture, and high nutritional quality. Similar to their findings, our study confirms that xanthan gum effectively enhances the suspension stability of quinoa-based beverages by forming a robust physical network that prevents particle settling, thereby maintaining uniformity during storage. This supports the broad applicability of xanthan gum as a stabilizer in plant-based beverages to improve stability and sensory attributes.

### 3.11 Sensory attributes

In order to evaluate the impact of pretreatments on final quinoa beverages, a sensory analysis was firstly performed without addition of food additives. The results in Table 1S showed that pretreatments and technological processing improved the sensory perception of quinoa beverages-like milk, but not enough for panelists' acceptability. The panelists generated typical descriptors for all treated quinoa beverages such as bitter after taste, green flavor, vegetal aroma, leguminous taste, and nutty flavor.

Finally, quinoa beverages infused with natural flavorings, such as guava and vanilla, exhibited significantly higher sensory attributes compared to the products without these additives. The changes in sensory perception, including the presence of fruit taste and natural aroma, can be attributed to the reduction or masking of off-flavors, as well as the introduction of new tastes and odors that help improve the overall sensory experience and mask any sensory defects (Table 11). Among raw and treated quinoa beverages, only soaked quinoa beverage followed by malted and germinated have high acceptable scores by panelist which recorded sensorial score 7.1 ± 0.5 (Like moderately), 6.5 ± 1.0 (Like slightly/Like moderately), and 6.3 ± 0.7 (Like slightly) for soaked, malted, and germinated quinoa beverage respectively. These outcomes confirmed with our data in Table 1 that stated that the aqueous soaking process led to obtain free saponin of quinoa-based foods.

**Table 11:** Sensory score of quinoa beverage emulsion with vanilla as odor and guava as flavor*.

Quinoa Beverage	Color	Aroma	Taste	Mouthfeel	Overall Acceptability
Raw	6.1 ± 1.6^b^	6.0 ± 1.3^b^	5.9 ± 0.7^a^	6.8 ± 1.2^ab^	5.8 ± 1.7^b^
Soaked	7.5 ± 0.5^a^	7.7 ± 0.5^a^	6.6 ± 1.7^a^	7.0 ± 0.9^a^	7.1 ± 0.5^a^
Germinated	7.0 ± 0.6^ab^	6.0 ± 0.8^b^	6.1 ± 1.0^a^	6.7 ± 1.8^ab^	6.3 ± 0.7^ab^
Malted	6.3 ± 1.6^b^	7.0 ± 1.5^a^	6.6 ± 1.0^a^	6.8 ± 1.0^ab^	6.5 ± 1.0^ab^
Dehulled	6.2 ± 0.8^b^	5.7 ± 0.9^b^	5.7 ± 0.7^a^	5.7 ± 0.8^c^	5.5 ± 1.3^b^

Mean values with the different superscripts in the same column indicate a significant difference at p ≤ 0.05.

## 4 The limitations & recommendations

For industrial relevance, extended shelf-life studies of at least 21 days, including microbial and physicochemical assessments, are essential and should be pursued in future research. Although the laboratory-scale ultrasonication parameters (20 kHz, 750 W) demonstrated effectiveness in improving product stability, scaling up this technology requires careful consideration of energy input, equipment costs, and processing throughput. Compared to conventional homogenization, ultrasonication may offer advantages such as reduced processing time and enhanced product quality; however, comprehensive techno-economic analyses are necessary to evaluate its cost-effectiveness and industrial feasibility. Furthermore, future studies should incorporate HPLC or LC-MS validation of saponin content, especially in formulations intended for commercial or nutritional health claims.

## 5 Conclusions

In recent years, consumers' demand for quinoa-based foods has surged due to their health benefits and nutritional value. However, widespread adoption remains constrained by the low stability and suboptimal sensory attributes of quinoa beverages. To address these challenges, various pretreatment methods have been explored. Ultrasonication was employed as an advanced processing technique to enhance the stability of quinoa beverages. Additionally, the impact of incorporating hydrocolloids i.e., Arabic gum, xanthan gum, and psyllium husk beside natural flavors (aroma and taste) with vanilla and guava fruit were examined in the current study to enhance the quality of the final product. The findings demonstrated that bioprocessing techniques—such as aqueous soaking, malting, dehulling, and germination—significantly improved the nutritional profile of quinoa beverages. These treatments enhanced phenolic compounds, flavonoids, antioxidant capacity with reducing saponin content, which is known to contribute to bitterness. Sensory evaluations indicated a marked reduction in undesirable off-flavors, particularly in soaked, malted, and germinated quinoa beverages, compared to raw or pearled quinoa beverages. Moreover, hydrocolloids and natural flavors played a crucial role in improving the beverage's texture, mouthfeel, and overall consumer appeal while extending shelf stability during refrigerated storage. From a commercial perspective, these findings present significant opportunities for the functional beverage market. By leveraging bioprocessing and formulation strategies, food manufacturers can develop quinoa-based beverages with superior stability, enhanced nutritional value, and improved sensory appeal—factors that are key to consumer acceptance and market success. Given the rising demand for plant-based, nutrient-dense drinks, these innovations could position quinoa beverages as a competitive alternative choice in beverage technology as a health-promoting beverage. This study provides a suitable and easy-to-use foundation for scaling up, allowing food and beverage companies to introduce innovative, high-quality quinoa beverages that meet consumer expectations and align with industry trends in health-conscious consumption. Additionally, while the ultrasound-assisted technique demonstrated promising results at lab scale, further optimization is needed to enable industrial-scale application. Alternative food-grade hydrocolloids, could be explored, and long-term storage studies may be conducted in the future to evaluate potential antimicrobial effects and natural preservatives.

## CRediT authorship contribution statement

**Sobhy A. El-Sohaimy:** Writing – review & editing, Visualization, Validation, Supervision, Project administration, Conceptualization. **Taha Mehany:** Writing – review & editing, Writing – original draft, Validation, Software, Methodology, Investigation, Funding acquisition, Formal analysis, Data curation. **Mohamed G. Shehata:** Writing – review & editing, Visualization, Investigation, Formal analysis, Conceptualization. **Ashraf A. Zeitoun:** Validation, Supervision. **Hanan M. Alharbi:** Software, Investigation, Formal analysis. **Khairiah Mubarak Alwutayd:** Visualization, Investigation, Formal analysis. **Mohamed A.M. Zeitoun:** Writing – review & editing, Supervision.

## Declaration of competing interest

The authors declare that they have no known competing financial interests or personal relationships that could have appeared to influence the work reported in this paper.

## Data Availability

Data will be made available on request.

## Appendix A Supplementary data

Supplementary material Table 1S. Sensory score of quinoa beverage emulsion without food additives. Fig. S1. Representative flow chart of quinoa seeds preparation and biochemical processing for enhanced functional and nutritional properties. Fig. S2. Foaming capacity of treated quinoa seeds, Where R: Raw; S: Soaked; G: Germinated; M: Malted; D: Dehulled. Fig. S3. Emulsifying activity of treated quinoa seeds. Where, R: Raw; S: Soaked; G: Germinated; M: Malted; D: Dehulled. Supplementary material
