# Supplementary material for: Optimizing functional, stability, and sensory attributes of quinoa beverages through bioprocessing, ultrasonication, and hydrocolloids
Source: Food Chem X. 2025 Aug 18;30:102923. doi: 10.1016/j.fochx.2025.102923 (PMC12397925; doi:10.1016/j.fochx.2025.102923)
Supplement: Supplementary material — Table 1S. Sensory score of quinoa beverage emulsion without food additives. Fig. S1. Representative flow chart of quinoa seeds preparation and biochemical processing for enhanced functional and nutritional properties. Fig. S2. Foaming capacity of treated quinoa seeds, Where R: Raw; S: Soaked; G: Germinated; M: Malted; D: Dehulled. Fig. S3. Emulsifying activity of treated quinoa seeds. Where, R: Raw; S: Soaked; G: Germinated; M: Malted; D: Dehulled. [file mmc1.docx]

***Supplementary data for:***

***–––––––––––––––––––––––––––––––––––––––––––––––––––––––––––––––––––––––––***

**Article**

**Optimizing functional, stability, and sensory attributes of quinoa beverages through bioprocessing, ultrasonication, and hydrocolloids**

Sobhy A. El-Sohaimy ^a,b, †^, Taha Mehany ^a, †,*^, Mohamed G. Shehata ^a,c^, Ashraf A. Zeitoun ^d^, Hanan M. Alharbi ^e^, Khairiah Mubarak Alwutayd ^e^, Mohamed A. A. Zeitoun ^d^

^a^ Food technology Department, Arid Lands Cultivation Research Institute, City of Scientific Research and Technological Applications, 21934 Alexandria, Egypt

^b^ Department of Technology and Organization of Public Catering, Institute Sport, Tourism and Service, South Ural State University, 454080 Chelyabinsk, Russian Federation

^c^ Food Research Section, Applied Research and Capacity Building Division, Abu Dhabi Agriculture and Food Safety Authority (ADAFSA), Abu Dhabi 20602, United Arab Emirates

^d^ Department of Food Science, Faculty of Agriculture (Saba Basha), Alexandria University, 21531Alexandria, Egypt

^e^ Department of Biology, College of Science, Princess Nourah bint Abdulrahman University, P.O. Box 84428, Riyadh 11671, Saudi Arabia

^†^ These authors contributed equally to this work.

[*tahamehany@yahoo.com](mailto:*tahamehany@yahoo.com)

Table 1S. Sensory score of quinoa beverage emulsion without food additives.

| **Quinoa Beverage** | **Color** | **Aroma** | **Taste** | **Mouthfeel** | **Overall Acceptability** |
| --- | --- | --- | --- | --- | --- |
| **Raw** | 5.4±2.5 ^b^ | 5.1±2.5 ^b^ | 5.0±1.7 ^ab^ | 5.0±2.3 ^b^ | 5.1±1.7 ^ab^ |
| **Soaked** | 7.5±0.5 ^a^ | 7.5±0.7 ^a^ | 6.4±1.2 ^a^ | 6.9±0.8 ^a^ | 6.8±0.6 ^a^ |
| **Germinated** | 6.8±0.9 ^ab^ | 5.6±0.8 ^ab^ | 5.3±1.1 ^ab^ | 5.1±1.2 ^b^ | 5.3±1.2 ^b^ |
| **Malted** | 6.0±0.8 ^ab^ | 5.3±0.9 ^ab^ | 5.3±0.9 ^ab^ | 5.0±0.6 ^b^ | 5.2±1.4 ^b^ |
| **Dehulled** | 5.7±2.4 ^b^ | 6.2±2.5 ^ab^ | 4.7±1.9 ^b^ | 5.7±2.1 ^ab^ | 5.0±2.3 ^b^ |

Mean values with the different subscripts in the same column indicate a significant difference at p ≤0.05.

Figure S1. Representative flow chart of quinoa seeds preparation and biochemical processing for enhanced functional and nutritional properties.


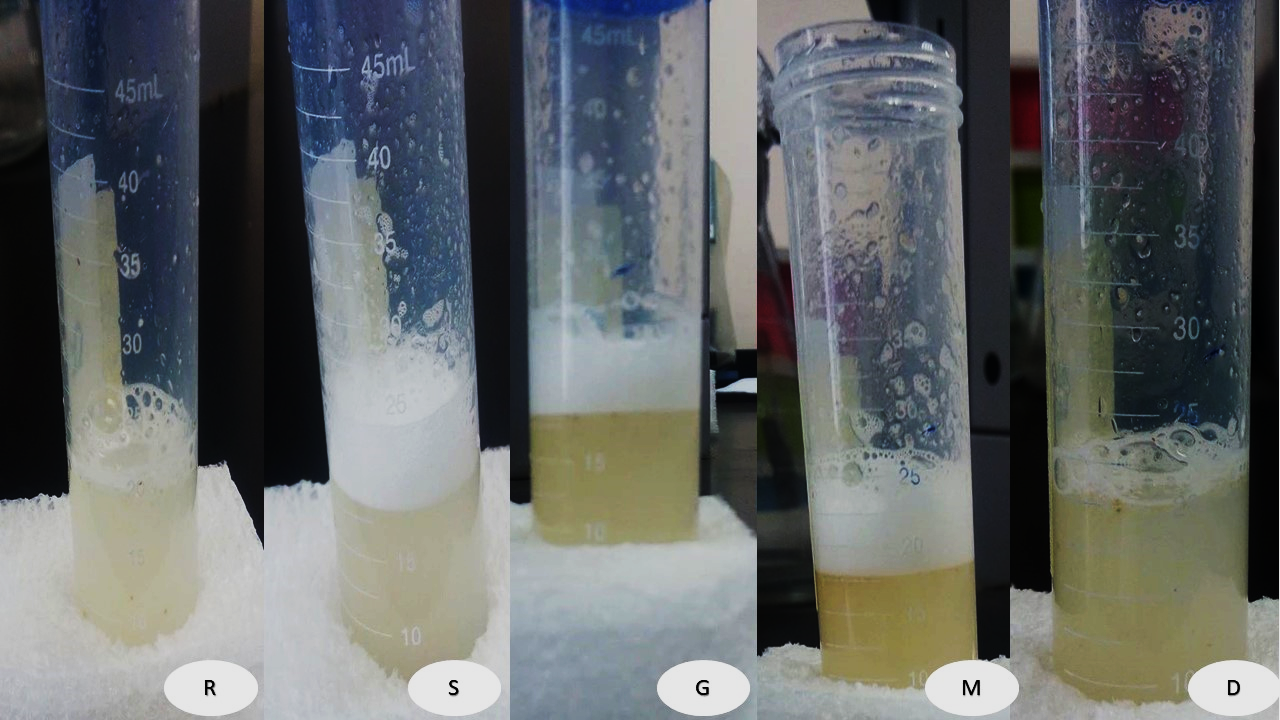


**Figure S2.** Foaming capacity of treated quinoa seeds, Where, R: Raw; S: Soaked; G: Germinated; M: Malted; D: Dehulled.


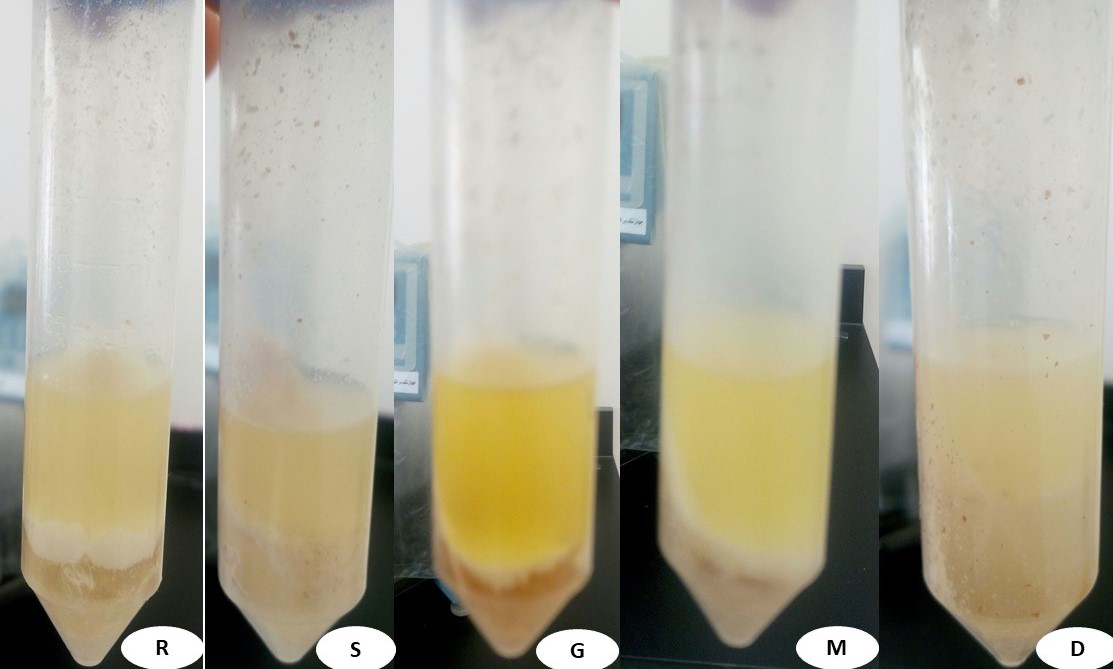


**Figure S3.** Emulsifying activity of treated quinoa seeds. Where, R: Raw; S: Soaked; G: Germinated; M: Malted; D: Dehulled.
